# Short-term effects of weather and air pollution on atopic dermatitis symptoms in children: A panel study in Korea

**DOI:** 10.1371/journal.pone.0175229

**Published:** 2017-04-06

**Authors:** Young-Min Kim, Jihyun Kim, Youngshin Han, Byoung-Hak Jeon, Hae-Kwan Cheong, Kangmo Ahn

**Affiliations:** 1 Environmental Health Center for Atopic Diseases, Samsung Medical Center, Seoul, Korea; 2 Department of Pediatrics, Samsung Medical Center, Sungkyunkwan University School of Medicine, Seoul, Korea; 3 Department of Social and Preventive Medicine, Sungkyunkwan University School of Medicine, Suwon, Korea; National Yang-Ming University, TAIWAN

## Abstract

**Introduction:**

The effects of weather and air pollution on the severity and persistence of atopic dermatitis (AD) are important issues that have not been investigated in detail. The objective of our study was to determine the short-term effects of meteorological variables and air pollution on AD symptoms in children.

**Methods:**

We enrolled 177 AD patients with 5 years or younger from the Seoul Metropolitan Area, Korea, and followed for 17 months between August 2013 and December 2014. Symptoms records of 35,158 person-days, including itching, sleep disturbance, erythema, dry skin, oozing, and edema, were obtained. We estimated the effect of meteorological variables including daily mean temperature, relative humidity (RH), diurnal temperature range (DTR), rainfall and air pollutants including particulate matter with an aerodynamic diameter ≤10 μm (PM_10_), nitrogen dioxide (NO_2_), and tropospheric ozone (O_3_) on AD symptoms using a generalized linear mixed model with adjustment for related confounding factors.

**Results:**

A 5°C increase in outdoor temperature and a 5% increase in outdoor RH was associated with 12.8% (95% confidence intervals (CI): 10.5, 15.2) and 3.3% (95% CI: 1.7, 4.7) decrease in AD symptoms, respectively, on the same day. An increase of rainfall by 5 mm increased AD symptoms by 7.3% (95% CI: 3.6, 11.1) for the days with <40 mm rainfall. The risk of AD symptoms increased by 284.9% (95% CI: 67.6, 784.2) according to a 5°C increase in DTR when it was >14°C. An increase in PM_10_, NO_2_, and O_3_ by 10 units increased the risk of AD symptoms on the same day by 3.2% (95% CI: 1.5, 4.9), 5.0% (95% CI: 1.4, 8.8), and 6.1% (95% CI: 3.2, 9.0), respectively.

**Conclusion:**

Exposure to meteorological variables and air pollutants are associated with AD symptoms in young children.

## Introduction

Atopic dermatitis (AD) is a chronic inflammatory skin disease mostly occurring in early childhood and the prevalence of AD in children is still increasing in both developing and developed countries [1, 2]. In Korea, a nationwide cross-sectional study in 2010 demonstrated that the prevalence of ‘itchy eczema, ever’ was 27.0% for children aged 6–7 years and 19.9% for adolescents aged 12–13 years [3]. Because of its high prevalence and progression into respiratory allergies [1], the management of ongoing AD is a critical issue in public health. One of the basic principles in the management of AD is to avoid aggravating factors; these may include microbes, allergens, emotional stress, meteorological factors and pollutants in both indoor and outdoor environments [1, 4, 5].

Concerns regarding the impact of climatic factors on atopic diseases are growing as climate change is now widely recognized as a major environmental problem [6–11]. One group found that the annual variation of temperature and relative humidity (RH) were negatively associated with both asthma and eczema symptoms in children [11]. The prevalence of eczema in the United States is lowest in parts of the country where there is abundant sun exposure, high humidity, and elevated temperatures [9]. In contrast, a prospective cohort study of American children found that poorly controlled eczema was influenced by long-term weather patterns, including high temperatures and increased sun exposure [12]. Most previous studies, however, evaluated long-term weather effect on AD. Few studies have focused on the short-term effects of weather on flares in children with preexisting AD. The acute or subacute effects of meteorological variables on the severity and persistence of eczema symptoms must be assessed to determine the appropriate management of AD.

Air pollutants are also considered as risk factors for AD. In a study involving 4,907 French children residing at their current address for 3 years or longer, lifetime eczema was significantly associated with 3-year averaged concentrations of particulate matter with an aerodynamic diameter ≤ 10 μm (PM_10_), nitrogen dioxide (NO_2_), and carbon monoxide (CO) [13]. Several studies have reported that exposure to outdoor and indoor air pollutants is associated with worsening AD [14–17]. In our previous study, elevated outdoor concentrations of PM_10_ were significantly associated with increased AD symptoms on the following day [14]. However, investigating the impact of air pollution on AD is still challenging. Moreover, there are few prospective studies that have evaluated the acute effect of air pollution on AD symptoms.

In this study, we investigated whether outdoor air pollution and meteorological variables including temperature, humidity, diurnal temperature range (DTR), and rainfall affect AD symptom flares in children with pre-existing AD by measuring daily symptom scores and exposure levels to outdoor environment.

## Methods

### Subjects and AD symptom assessment

A total of 177 children with AD (110 boys and 67 girls) were enrolled. They were infants and young children with the age of 5 years or younger who lived in the Seoul Metropolitan Area, Korea. The patients were followed for 17 months between August 2013 and December 2014. The diagnosis of AD was determined by two pediatric allergists according to the Hanifin and Rajka criteria [18]. The severity of AD was assessed using the SCORing Atopic Dermatitis (SCORAD) index [19]. The total IgE and specific IgE against common food and inhalant allergens in the peripheral blood were measured using ImmunoCAP (Thermo Fisher Scientific Inc., Waltham, MA, USA), and considered positive if >0.35 kU/L.

The parents were instructed to record AD symptoms on a daily basis. Parents used a smartphone-adjusted symptom diary that we developed to describe the extent of itching, sleep disturbance, erythema, dryness, oozing, and edema on a scale of 0 to 4. The presence of AD symptoms was determined when a symptom score was 2 points or more for both itching and sleep disturbance scores, accompanied by at least 2 points of the sum of the following symptoms: erythema, dryness, edema, or oozing. For the statistical analysis, the presence of AD symptoms was coded as a binary variable (0 or 1).

Written informed consent was obtained from the parents or guardians of all participating children. The study protocols were reviewed and approved by the Institutional Review Board (IRB) at Samsung Medical Center (IRB No. 2013-05-009).

### Meteorological variables and air pollution

Meteorological data including hourly outdoor temperature, hourly outdoor RH and daily rainfall were obtained from the Korean Meteorological Administration (KMA) which runs 76 automatic weather stations in the Seoul Metropolitan Area. Based on the hourly temperature data, we calculated the daily mean temperature. DTR was calculated as the difference between the daily maximum and minimum temperatures. The hourly concentrations for PM_10_, NO_2_, and tropospheric ozone (O_3_) were collected from the National Institute of Environmental Research, which monitors the ambient air pollution at 111 sites in the study area. The daily 24 hour-average values of PM_10_ and NO_2_, as well as the maximum daily 8 hour-average value of O_3_ were calculated. We matched the daily levels of meteorological variables and air pollutants with the daily AD symptom data based on residential address.

### Statistical analysis

Considering the repeated measurement of the AD symptoms based on the panel of AD patients, we adopted a generalized linear mixed model (GLMM) with binomially distributed errors to estimate the effects of the meteorological variables and air pollutants on the AD symptoms.

Ambient air pollution is strongly associated with daily variations in meteorology [20,21]. For example, O_3_ is a secondary air pollutant that strongly depends on meteorological conditions. Tai et al. [21] reported that 50% of particulate matter with an aerodynamic diameter of ≤2.5 μm (PM_2.5_) could be explained by temperature, RH, precipitation, and circulation in the United States. As a result, meteorological variables and air pollution may affect AD symptoms with interaction. All of the predicting variables, including meteorological variables and air pollutants were, therefore, included in a GLMM analysis. Age, sex, SCORAD at enrollment, the presence of fever (0 or 1, as a proxy of infection), and the day of week (DOW) were controlled in the analysis. We treated temperature, DTR, RH, rainfall, PM_10_, NO_2_, and O_3_ as fixed effects and each participant as a random effect in the models. The model specifications are as follows:

$$\begin{array}{l} LnE(Y)=\beta_{0}+\beta_{1}(Temp)+\beta_{2}(DTR)+\beta_{3}(RH)+\beta_{4}(Rain)+\beta_{5}(PM_{10})+\beta_{6}(NO_{2})+\beta_{7}(O_{3})\\ +SCORAD+factor(fever)+factor(sex)+factor(DOW)+\gamma (subject) \end{array}$$ (1)

where *E(Y)* is the expected expression of AD symptoms; *Temp* is daily mean temperature; *DTR* is diurnal temperature range; *RH* is relative humidity; *Rain* is rainfall; *PM*_*10*_ is particulate matter with an aerodynamic diameter ≤ 10 μm; *NO*_*2*_ is nitrogen dioxide, and *O*_*3*_ is tropospheric ozone; *SCORAD* is the SCORing Atopic Dermatitis index at enrollment; *fever* is the presence of fever on the day of record; *DOW* is the day of week; and *γ* is the random effect for subjects.

We also used penalized regression curves of a generalized additive mixed model (GAMM) to examine the linearity of the relationship between the meteorological variables and air pollutants with AD symptoms [22]. Like the GLMM, all of the predicting variables were included in the GAMM analysis, adopting smoothing splines with adjustment for the same confounding factors as the GLMM.

Moving averages (MAs) up to 6 days of meteorological variables and air pollution were used to examine the cumulative effects of weather and air pollution on AD symptoms. For instance, the MA0-5 of PM_10_ indicates an average concentration of PM_10_ over 6 days (including the previous 5 days and the current day). We also analyzed the effects of meteorological variables and air pollution on AD symptoms according to sex. The percent change in risk and 95% confidence intervals (CI) were calculated using a regression coefficient. These factors were measured according to 5-unit increases in temperature (°C), RH (%), DTR (°C), rainfall (mm/day) and10-unit increases in PM_10_ (μg/m^3^), O_3_ (ppb) and NO_2_ (ppb).

All procedures were conducted using the R version 3.0.0 (The Comprehensive R Archive Network: http://cran.r-project.org) with the “lme4” package (version 3.1–2) for GLMM and “mgcv” package (version1.8–3) for GAMM model fitting. All tests were two-sided. An alpha level <0.05 was considered significant.

## Results

During the 17 months of study period, the symptom records of 35,158 person-days were obtained. A total of 23,454 (66.7%) and 11,704 (33.3%) person-days were recorded by boys and girls, respectively. The average age of the study subjects was 2.0 years. AD symptoms were present in 49.9% of boys and 39.4% of girls (*p* < .0001). The average SCORAD at the time of enrollment was 31.1 (Table 1).

**Table 1:** Characteristics of the study subjects and summary of atopic dermatitis symptoms^a^.

Characteristics	Total	Boys	Girls	*P*-value^b^
**No. of subjects**	177	110 (62.1%)	67 (37.9%)	
**Age (years)**	2.0 ± 1.6	1.8 ± 1.6	2.3 ± 1.7	0.085
**Height (cm)**	83.9 ± 14.7	82.8 ± 14.6	85.5 ± 14.8	0.231
**Weight (kg)**	11.9 ± 4.0	11.8 ± 4.1	11.9 ± 3.7	0.440
**SCORAD**^c^	31.1 ± 12.8	31.2 ± 13.4	31.0 ± 11.9	0.951
**Total IgE (U/L)**	366.4 ± 801.2	374.7 ± 844.8	350.0 ± 727.0	0.551
**Sensitization** (%)				
**Food allergens**^d^	45.2	46.5	43.1	0.762
**Inhalant allergens**^e^	62.1	58.8	66.7	0.838
**Presence of fever (%)**	2.9	2.9	2.9	0.976
**No. of record (person-days)**	35,158	23,454 (66.7%)	11,704 (33.3%)	
**Symptom presence (%)**	44.1	49.9	39.4	< .0001

^a^ Data are expressed as mean ± standard deviation;

^b^Test for differences between boys and girls: t-test for means of each variable, except symptom presence, which was compared using the Mann-Whitney U test;

^c^ SCORAD (SCORing of Atopic Dermatitis) index at enrollment;

^d^ Sensitized by five allergens, including egg white, cow’s milk, soybean, wheat, and peanut;

^e^ Sensitized by house dust mite (*Dermatophagoides pteronyssinus*, *D*. *farinae*).

The average daily mean temperature was 15.0°C and RH was 64.9% (Table 2). The average values of DTR and rainfall were 8.9°C and 2.7 mm/day, respectively. The mean concentrations of PM_10_, NO_2_ and O_3_ were 45.2 μg/m^3^, 32.4 ppb and 38.1 ppb, respectively.

**Table 2:** Summary of meteorological variables and air pollutant levels during the study period.

Variable		Mean ± SD^a^	Minimum	Maximum
**Meteorological variable**	**Temperature (°C)**	15.0 ± 9.7	-11.2	32.0
	**DTR**^b^ **(°C)**	8.9 ± 3.0	1.6	22.2
	**Relative humidity (%)**	64.9 ± 14.3	26.0	100.0
	**Rainfall (mm/day)**	2.7 ± 9.7	0.0	157.5
**Air pollutant**	**PM**_**10**_ **(μg/m**^**3**^**)**	45.2 ± 26.4	3.6	193.7
	**NO**_**2**_ **(ppb)**	32.4 ± 13.4	1.0	104.5
	**O**_**3**_ **(ppb)**	38.1 ± 20.3	1.1	123.0

^a^SD: standard deviation;

^b^ DTR: diurnal temperature range.

**Fig 1:**
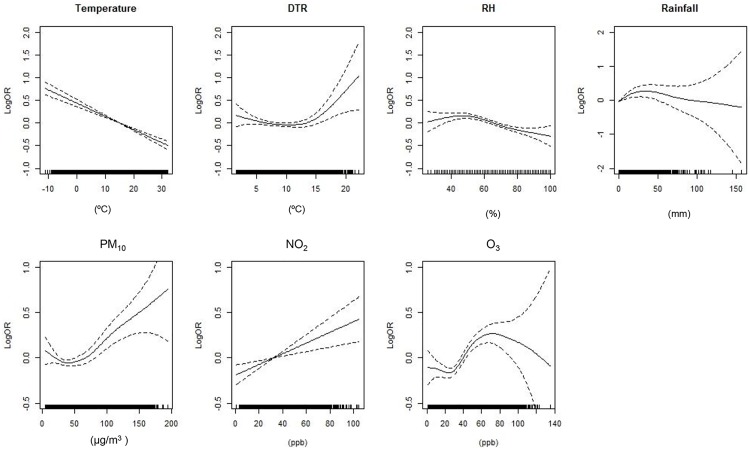
Relationship of meteorological variables and air pollution with Atopic Dermatitis (AD) symptoms. Each figure shows the spline curve (solid lines) with a 95% confidence intervals (two dashed lines). All variables are based on the same model on the same day, without using moving averages for the exposure to each risk factor. The model was controlled for the severity score at the initial visit, age, sex, presence of fever, and day of week (DOW). RH: relative humidity; DTR, diurnal temperature range; OR: odds ratio.

The spline curves shown in Fig 1 demonstrate the relationship of meteorological variables and air pollution with the presence of AD symptoms on the same day (MA0). These results were obtained using GAMM fitting, controlling for SCORAD at enrollment, age, sex, fever, and DOW. The outdoor temperature showed negative linear relationships with AD symptoms. In contrast, the relationships of RH, rainfall, and DTR with AD symptoms were nonlinear. Interestingly, DTR and AD symptoms showed a positive relationship when DTR was above 14°C and daily rainfall was also positively related with AD symptoms when rainfall was less than approximately 40 mm/day. There was a negative relationship between RH and AD symptoms overall although the relationship was not linear.

NO_2_ showed positively linear relationship with AD symptoms for the whole range, suggesting that it has hazardous effects on AD. PM_10_ had positive relationship with AD symptoms when it was approximately over 35 μg/m^3^. Relationship between O_3_ and AD symptoms was not linear. However, the relationship was linear within the range of 30–70 ppb of O_3_ concentration.

**Fig 2:**
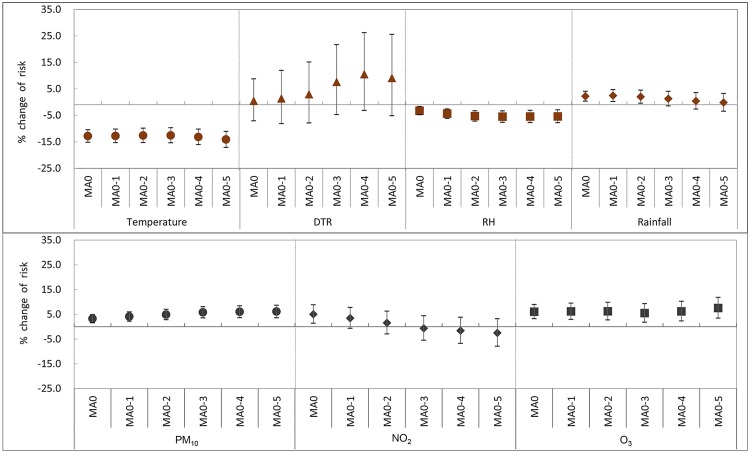
Effects of meteorological variables and air pollution on Atopic Dermatitis (AD) symptoms by moving average. Data represent percent changes and 95% confidence intervals in AD symptoms per 5-unit increase in daily mean temperature (°C), relative humidity (%), diurnal temperature range (°C) and 10-unit increase in PM_10_ (μg/m^3^), NO_2_ (ppb), and O_3_ (ppb). RH: relative humidity; DTR: diurnal temperature range; MA: moving average.

Fig 2 shows the effect of meteorological variables and air pollution on AD symptoms as a result of GLMM fitting after controlling for SCORAD at enrollment, age, sex, fever, and DOW. An increase in daily mean temperature by 5°C was significantly associated with a 12.8% (95% CI: 10.5, 15.2) decrease in AD symptoms on the same day (Table 3). A statistically significant association was also found between a decrease in AD symptoms and an increase in various moving averaged temperatures. Similarly, an increase in outdoor RH by 5% decreased the risk of AD symptoms from 3.3% (95% CI: 1.7, 4.8) with MA0 to 5.4% (95% CI: 2.9, 7.8) with MA0-5. This finding reflects the protective effect of elevated outdoor RH on AD. The effect was stronger as the number of moving average days increased, indicating that there was a lag effect. An increase in DTR of MA0-4, i.e. moving average of the previous 4 days and the current day, by 5°C increased AD symptoms by 10.6% (95% CI: -3.1, 26.2), but not significantly. Of note, we found that AD symptoms increased by 284.9% (95% CI: 67.6, 784.2) per 5°C increase in DTR when we fitted for the data >14°C DTR in the GLMM after controlling for the same confounders. A daily rainfall increase by 5 mm/day was significantly associated with an increase in AD symptoms by 2.2% (95% CI: 0.4, 4.1) on the same day. When we fit the same model for the daily rainfall data <40 mm/day, the effect size of rainfall increased to 7.3% (95% CI: 3.6, 11.1) per 5 mm increase in daily rainfall. However, the adverse effect of rainfall was not significant in the moving averages of daily rainfall after 2 days (MA0-2 through MA0-5).

**Table 3:** Percent changes of atopic dermatitis symptoms caused by meteorological variables and air pollution on the same day^a^.

Variable	Both	Boys	Girls
**Temperature (°C)**	-12.8 (-15.2, -10.5)*	-14.0 (-16.9, -11.0)*	-10.9 (-14.8, -6.9)*
**DTR**^b^ **(°C)**	0.5 (-7.1, 8.8)	-3.0 (-12.2, 7.1)	6.2 (-6.8, 21.0)
**Relative humidity (%)**	-3.3 (-4.8, -1.7)*	-3.0 (-4.9, -1.0)*	- 3.7(-6.2, -1.2)*
**Rainfall (mm/day)**	2.2 (0.4, 4.1)*	2.3 (-0.0, 4.6)	2.0 (-1.1, 5.2)
**PM**_10_ **(μg/m^3^)**	3.2 (1.5, 4.9)*	1.9 (-0.2, 4.0)	5.2 (2.5, 8.0)*
**NO_2_ (ppb)**	5.0 (1.4, 8.8)*	9.2 (4.4, 14.3)*	-1.1 (-6.5, 4.6)
**O_3_ (ppb)**	6.1 (3.2, 9.0)*	10.2 (6.4, 14.1)*	-0.1 (-4.3, 4.4)

^a^Data are expressed as percent changes and 95% confidence intervals. Percent change indicates a change in AD symptoms according to an increase of 5-units in daily mean temperature, relative humidity, DTR, rainfall and an increase of 10-unit in PM_10_, NO_2_ and O_3_;

^b^DTR: diurnal temperature range. All results were from the whole range of air pollution.

*p value < 0.05.

The concentrations of ambient PM_10_, NO_2_, and O_3_ were significantly associated with AD symptoms. An increase in the PM_10_ concentration by 10 μg/m^3^ increased the risk of AD symptoms by 3.2% (95% CI: 1.5, 4.9) with MA0 and 6.1% (95% CI: 3.6, 8.6) with MA0-5 for the whole range of PM_10_ concentration. When we fit the GLMM model for data with PM_10_ concentration over 35 μg/m^3^ (Fig 1), the risk of AD symptoms increased by 3.6% (95% CI: 1.5, 5.7) as PM_10_ increased by 10 μg/m^3^ on the same day, which is slightly higher than that for the whole range. The effects of the moving averages of PM_10_ from MA0 to MA0-5 on AD symptoms were statistically significant. This effect gradually increased as the number of averaging days increased. Similarly, the O_3_ concentration was positively associated with AD symptoms for all moving average levels of O_3_ (MA0 to MA0-5). Considering the relationship between O_3_ and AD symptoms was linear within the range of 30–70 ppb of O_3_ concentration (Fig 1), we selected data within this range. When the GLMM was fitted and the same confounders were controlled, the AD symptoms increased by 9.1% (95% CI: 3.6,14.9) per 10 ppb increase in O_3_ concentration. This effect was greater than 6.1% (95% CI:3.2, 9.0) for the whole range (Table 3). An increase in NO_2_ concentration by 10 ppb increased the risk of AD symptoms on the same day by 5.0% (95% CI: 1.4, 8.8) and there was no lag effect of NO_2_ on AD symptoms.

**Fig 3:**
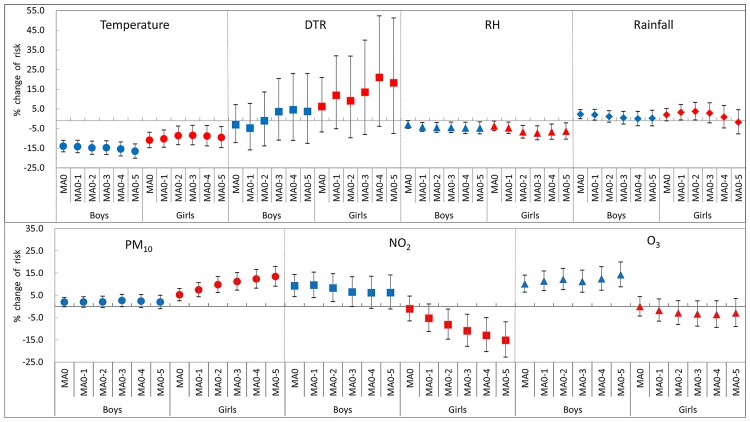
Effects of meteorological variables and air pollution on Atopic Dermatitis (AD) symptoms in boys (A) and girls (B). Data represent percent changes and 95% confidence intervals in AD symptoms per 5-unit increase in daily mean temperature (°C), relative humidity (%), diurnal temperature range (°C), and rainfall (mm) and 10-unit increase in PM_10_ (μg/m^3^), NO_2_ (ppb), and O_3_ (ppb). DTR: diurnal temperature range; RH: relative humidity; MA: moving average.

Fig 3 shows the effects of meteorological variables and air pollution on AD symptoms by sex. In both boys and girls, the positive association of temperature with AD symptoms was found. A similar result was observed in RH (Table 3). There was a significantly harmful effect of PM_10_ in girls, who showed a 5.2% (95% CI: 2.5, 8.0) change per 10 μg/m^3^ change in PM_10_ on the same day (MA0), whereas there was no such significant effect in boys. Boys exhibited 9.2% (95% CI: 4.4, 14.3) and 10.2% (95% CI: 6.4, 14.1) increases in AD symptoms by 10 ppb increase in NO_2_ and O_3_ concentration, respectively. However, there was no significant association of NO_2_ and O_3_ with AD symptoms in girls. These divergent effects between boys and girls more clearly observed when the moving average levels (MA0 to MA0-5) were fitted to the GLMM (Fig 3).

## Discussion

Our results demonstrated that short-term exposure to meteorological factors and air pollution are strongly associated with AD symptoms in children. Among four meteorological factors, increases in outdoor temperature and RH significantly reduced AD symptom scores. In contrast, increases in daily rainfall and DTR aggravated AD symptoms. Ambient PM_10_, NO_2_, and O_3_ increased the risk of AD symptoms. The effects of PM_10_ and O_3_ increased as the number of days of moving average increased, indicating that there were lag effects.

Our finding of negative associations between outdoor temperature and humidity and AD symptoms is consistent with a previous study in which a dry environment increased mast cell numbers and histamine content in the dermis of hairless mice [23]. Lower temperature typically results in greater use of indoor heating and lower indoor humidity, both of which aggravate eczema symptoms [9]. Consequently, our result supports the recommendation that outdoor temperature and humidity should be considered to alleviate AD symptoms, although the evidence level is not strong [24]. In contrast, several studies have reported opposing results. Langan et al. [25] found that increased humidity and heat were associated with a higher incidence of disease flares in a 28-day observational study of 25 Irish children. In a prospective cohort study of American children, Sargen et al. [12] demonstrated that warm and humid climates are associated with poorly controlled eczema. The associations between weather and AD symptoms might vary according to region and season. More studies are required to elucidate the role of temperature and humidity in AD.

DTR as well as the absolute level of ambient temperature influences our health. DTR has an adverse effect on mortality and morbidity in cardiovascular and respiratory diseases [26–29]. An increase in DTR >10°C was associated with increased emergency room admissions in asthmatic children [29]. However, the relationship between daily temperature variation and AD was not reported so far. In this study, we found that AD symptoms increased by 284.9% (95% CI: 67.6, 784.2) per 5°C increase in DTR when it was >14°C. These results suggest that children with AD should be more careful on days with elevated DTR, even on the days with high daily mean temperatures. To the best of our knowledge, this is the first study addressing the adverse effect of daily temperature fluctuation on AD symptom.

The Intergovernmental Panel on Climate Change (IPCC) [30] projected that in the future, extreme weather patterns (including cold/hot temperatures, drought, and flood) will increase in frequency as a result of climate change, particularly in temperate regions. According to a report on future climate change in the Korean peninsula published by the KMA [31], the average annual precipitation of the Korean Peninsula will exceed the natural variation and show a clear increase after the mid-21st century. This change is projected to occur in both representative concentration pathways (RCP) 4.5 and RCP 8.5, which are climate change scenarios adopted by the IPCC since 2011. This increase will be approximately 3.9 times the global average. It implies that the adverse effect of rainfall on AD symptoms found here would increase in the future, leading to poor control of preexisting AD symptoms.

Based on the large data set derived from daily symptom records, we demonstrated that air pollutants, including PM_10_, NO_2_, and O_3_, are related to the aggravation of AD symptoms; this finding supports those of previous studies [14, 15, 32]. The effects of PM_10_ and O_3_ were robust; the effect size increased as the MA0 went up to MA0-5 (Fig 2). Of interest, we found that the ambient O_3_ concentration had a greater effect on AD symptoms in children who were exposed within the range of 30–70 ppb than over the entire range. Although the mechanism is not clearly understood, behavioral changes might be involved. There is a possibility that patients with AD tried to avoid exposure to O_3_ when high levels were detected and announced by the local government. As O_3_ will increase by the climate change, particularly during warm regions such as the Korean peninsula [33–36], it is necessary to reduce O_3_ level for the management of AD.

Another interesting finding in this study was that there were differences in the harmful effect of air pollutants on AD symptoms according to sex. There was a significantly harmful effect of PM_10_ on AD symptoms in girls, whereas NO_2_ and O_3_ in boys. With regards to AD, it has been known that there are gender differences in prevalence and quality of life [37–39]. In addition, immune development and behavioral patterns such as outdoor play are different between boys and girls [40–42]. Infection and gut microbiota affects males and females differently [43]. However, there is no evidence to explain the sex-specific response to air pollutants. Although boys have a different immune development in early childhood compared to girls, leading to a higher sensitization rate, total IgE level and peripheral eosinophil counts [40], the exact reason needs to be investigated.

In this study, we analyzed the relationships between AD symptoms and weather, and air pollution using GAMM and GLMM. GAMM contains a random effect, in addition to penalized spline curves, which the generalized additive model (GAM) contains. GAM and GAMM allow for nonparametric adjustments to nonlinear confounding effects on trends and other factors, such as weather variables [44, 45]. GAMM is appropriate to determine whether relationships are linear and, if so, whether positive or negative. However, GAM and GAMM are not used to estimate the effect sizes of risk factors because they are nonparametric models. In contrast, GLMM is an extension to the generalized linear model, while containing random effects in addition to the usual fixed effects [46]. To estimate the effect size of the risk factors, we adopted a panel study using daily-repeated measurements for AD symptoms and environmental variables. This is the reason why we adopted two statistical models for data analysis in our study. GAMM was used to determine whether the relationships were linear or not, and GLMM to estimate the effect sizes of the risk factors on AD symptoms.

One of the strengths of this panel study is that we collected individual symptom records on a daily basis using smartphone-adjusted symptom diary. We also analyzed a large data set of 35,158 person-days by fitting GAMM and GLMM after controlling for potential confounders. By these efforts, we were able to obtain more objective and reliable results.

However, our study also has several limitations. For instance, socioeconomic factors such as household income and parental education levels were not controlled in the models. Although the study participants were enrolled in a single center and therefore their socioeconomic statuses were likely similar, it is important to consider such confounding factors in the analysis. Another limitation is that we did not evaluate the effect of indoor environment. It is well known that children spend most of their time indoors. If we, therefore, consider indoor environment in the exposure assessments, our results about the air pollutants and weather may be different from the current results. In case of air pollution, previous studies addressed that indoor environment such as PM_10_ and NO_2_ in Seoul are very dependent on outdoor condition [47]. For example, indoor to outdoor ratios for PM_10_ and NO_2_ were 0.69 and 1.05, respectively [47–48]. This indicates that even if we considered indoor levels for PM_10_ and NO_2_, the effect of these factors would be similar as shown in the present study. In contrast, indoor temperature and RH are quite different from outdoor temperature and RH. If indoor levels of temperature and RH were controlled for the analysis of the present study, our results would be different. Further studies on the relationships between AD symptoms and indoor environmental factors including indoor temperature and RH are required.

## Conclusions

This study demonstrates that the short-term exposure to ambient temperature, RH, DTR, and rainfall are significantly associated with AD symptoms in infants and young children living in a temperate region. Low ambient temperature and RH, large DTR, and high rainfall may aggravate AD symptom in children. In addition, PM_10_, NO_2_, and O_3_ showed significantly adverse effects on AD symptoms with gender differences. These findings could be applied to assist in establishing a management strategy of AD symptoms such as early warning system based on weather and air quality forecasts.

## Abbreviations

AD: atopic dermatitis
CI: confidence interval
CO: carbon monoxide
DOW: day of week
DTR: diurnal temperature range
GAMM: generalized additive mixed model
GLMM: generalized linear mixed model
IPCC: Intergovernmental Panel on Climate Change
KMA: Korean Meteorological Administration
MA: Moving average
NO_2_: nitrogen dioxide
O_3_: tropospheric ozone
PM_10_: particulate matter with an aerodynamic diameter ≤ 10 μm
PM_2.5_: particulate matter with an aerodynamic diameter ≤ 2.5 μm
RCP: representative concentration pathways
RH: relative humidity
SCORAD: SCORing Atopic Dermatitis index
